# Risk Factors for COVID‐19 Infections Among Vaccinated Patients with Dementia: A Nationwide Population‐Based Cohort Study in Taiwan

**DOI:** 10.1002/alz70860_098839

**Published:** 2025-12-23

**Authors:** Chih‐Ching Liu, Chien‐Hui Liu

**Affiliations:** ^1^ National Taipei University of Nursing and Health Sciences, No. 365, Ming‐te Rd., Peitou Dist., Taipei, Taiwan; ^2^ National Yang Ming Chiao Tung University, Institute of Biomedical Informatics, Taipei, Taiwan

## Abstract

**Background:**

While the association between dementia and the prevalence of COVID‐19 has been established, limited evidence exists regarding the risk factors—including sociodemographic characteristics and clinical conditions—associated with COVID‐19 infections in vaccinated patients with dementia, particularly in Asia. This study aims to identify the sociodemographic factors and pre‐existing medical conditions that increase the risk of COVID‐19 infections among vaccinated patients with dementia in Taiwan.

**Method:**

In this retrospective population‐based cohort study, we identified 325,697 individuals aged 40 years or older with dementia from the Taiwan National Health Insurance Research Database (NHIRD) between January 1, 2009, and January 1, 2020. COVID‐19 vaccination status and infections were identified using data from the Taiwan Centers for Disease Control's COVID‐19 Vaccination Registry (2021–2022) and COVID‐19 Confirmation Registry (2020–2022). We explored sociodemographic factors (age, gender, low‐income status, urbanization level) and pre‐existing medical conditions that may influence the risk of new‐onset COVID‐19 infections using Cox regression analysis. These medical conditions included the Charlson Comorbidity Index, specific mental health comorbidities (e.g., bipolar disorder, major depressive disorder, and schizophrenia), and chronic diseases (e.g., diabetes, hypertension, stroke, chronic kidney disease, chronic obstructive pulmonary disease, and Parkinson's disease).

**Result:**

The study included 217,548 participants who received COVID‐19 vaccination prior to infection. The risk of COVID‐19 infections was significantly higher among vaccinated patients with dementia who were male, of older age, had lower income, lived in areas with higher urbanization levels, had higher Charlson Comorbidity Index scores, or had any of the specific medical conditions examined in this study.

**Conclusion:**

Vaccinated patients with dementia face an elevated risk of COVID‐19 infections, with associated factors differing according to sociodemographic and clinical characteristics. Public health interventions should be tailored to address these variations to effectively mitigate COVID‐19 infection risks in this population.

